# OMCD: OncomiR Cancer Database

**DOI:** 10.1186/s12885-018-5085-z

**Published:** 2018-12-06

**Authors:** Aaron L. Sarver, Anne E. Sarver, Ce Yuan, Subbaya Subramanian

**Affiliations:** 1 Institute of Health Informatics, University of Minnesota, Minneapolis, MN USA; 2 Department of Surgery, University of Minnesota, 11-212 Moos Tower Mayo Mail Code 195 420 Delaware Street SE, Minneapolis, MN 55455 USA; 3 Bioinformatics and Computational Biology, University of Minnesota, Minneapolis, MN USA; 4 Masonic Cancer Center, University of Minnesota, Minneapolis, MN USA

**Keywords:** Cancer, microRNA, OncomiR, TCGA, Database, miRNA, miRNA expression profile

## Abstract

**Background:**

microRNAs (miRNAs) are crucially important in the development of cancer. Their dysregulation, commonly observed in various types of cancer, is largely cancer-dependent. Thus, to understand the tumor biology and to develop accurate and sensitive biomarkers, we need to understand pan-cancer miRNA expression.

**Constructions:**

At the University of Minnesota, we developed the OncomiR Cancer Database (OMCD), hosted on a web server, which allows easy and systematic comparative genomic analyses of miRNA sequencing data derived from more than 9500 cancer patients tissue samples available in the Cancer Genome Atlas (TCGA). OMCD includes associated clinical information and is searchable by organ-specific terms common to the TCGA.

**Conclusions:**

Freely available to all users (www.oncomir.umn.edu/omcd/), OMCD enables (1) simple visualization of TCGA miRNA sequencing data, (2) statistical analysis of differentially expressed miRNAs for each cancer type, and (3) exploration of miRNA clusters across cancer types.

**Database URL:**

www.oncomir.umn.edu/omcd

## Background

microRNAs (miRNAs) are small noncoding RNAs that regulate posttranscriptional gene expression predominantly by binding to the 3′ untranslated region (UTR) of the target messenger RNAs [1]. Dysregulation of miRNAs has been associated with various types of cancer, such as colorectal cancer, lung cancer, lymphoma, glioblastoma, and osteosarcoma [2]. miRNA’s largely cancer-dependent dysregulation makes them candidate biomarkers for diagnosis, classification, and prognosis, as well as potential therapeutic targets [2]. Their use as biomarkers for diagnosis and classification has already been approved by the United States Food and Drug Administration (FDA) for lung, thyroid, and kidney cancer. miRNAs are also been approved by the FDA for identifying the primary site of other cancer types. To have a comprehensive understanding of the tumor biology and to develop accurate and sensitive biomarkers, we need comprehensive understanding of pan-cancer miRNA expression profiles.

The Cancer Genome Atlas (TCGA), a collaboration between the National Cancer Institute and the National Human Genome Research Institute, contains miRNA expression data for nearly 10,000 patients with 33 different cancer types [3]. Currently, the 2 major web-based repositories of analyzed TCGA data are the cBioPortal and the Broad Institute’s FireBrowse [4]. However, both of those platforms focus mainly on the analysis and visualization of genomic and mRNA expression data; neither of them enables in-depth analysis or comparative visualization of miRNA data. Still other databases, such as OncomiR, miRGator 3.0 and miRCancerdb enable analysis of TCGA miRNA data, calculate miRNA survival associations (OncomiR) or explore the miRNA-mRNA interactions (miRGator 3.0 and miRCacnerdb) [5–7]. These databases do not provide simple visualization of TCGA miRNA expression data or the ability to explore miRNA clusters.

At the University of Minnesota, we developed the OncomiR Cancer Database (OMCD), which enables (1) simple visualization of TCGA miRNA sequencing data, (2) statistical analysis of differentially expressed miRNAs for each cancer type, and (3) exploration of miRNA clusters across cancer types.

## Methods

To create OMCD, we used the LAMP software bundle (Linux, Apache 2, MySQL 5.0, and PHP) and Hypertext Markup Language (HTML), as described previously [8] and made the resulting website accessible to researchers across the globe. To host OMCD’s web application, we chose an Apache web server. To generate the user interface and enable communication with the MySQL database at the back end, we chose PHP, given its database-driven architecture that was designed for incorporation of additional information. Normalized expression data, statistical results, and annotation data are all stored in OMCD. To facilitate data retrieval and selection of different criteria for analysis, we designed a user-friendly graphic interface.

To construct the content of OMCD, we downloaded from TCGA the miRNA expression data of 9656 patients (represented by 8993 tumor samples and 663 control samples of normal tissue with 33 different cancer types (https://gdc.nci.nih.gov; Table 1). We used a 2-group *t* test to determine which miRNAs were differentially expressed between 1) control and tumor samples, for a given cancer type, 2) a cancer patient’s control sample, as compared with all other patients’ available control samples, and 3) a cancer patient’s tumor sample, as compared with all other patients’ available tumor samples. It can be noted that each of our 3 analyses had a different statistical power, which may account for the absence of a given miRNA from a specific dataset.

**Table 1:** Number of patients in the OncomiR Cancer Database (OMCD), by cancer type

Cancer Type (TCGA Code)	Total number of samples	Tumor	Normal
Breast invasive carcinoma [BRCA]	869	782	87
Brain Lower Grade Glioma [LGG]	530	530	0
Thyroid carcinoma [THCA]	573	514	59
Prostate adenocarcinoma [PRAD]	551	499	52
Ovarian serous cystadenocarcinoma [OV]	495	495	0
Head and Neck squamous cell carcinoma [HNSC]	532	488	44
Lung adenocarcinoma [LUAD]	504	458	46
Skin Cutaneous Melanoma [SKCM]	453	451	2
Uterine Carcinosarcoma [UCS]	450	418	32
Bladder Urothelial Carcinoma [BLCA]	436	417	19
Stomach adenocarcinoma [STAD]	450	404	46
Liver hepatocellular carcinoma [LIHC]	426	375	51
Lung squamous cell carcinoma [LUSC]	388	343	45
Cervical squamous cell carcinoma and endocervical adenocarcinoma [CESC]	313	310	3
Kidney renal papillary cell carcinoma [KIRP]	326	292	34
Colon adenocarcinoma [COAD]	280	272	8
Sarcoma [SARC]	263	263	0
Kidney renal clear cell carcinoma [KIRC]	332	261	71
Esophageal carcinoma [ESCA]	200	187	13
Pheochromocytoma and Paraganglioma [PCPG]	187	184	3
Pancreatic adenocarcinoma [PAAD]	183	179	4
Testicular Germ Cell Tumors [TGCT]	156	156	0
Thymoma [THYM]	126	124	2
Rectum adenocarcinoma [READ]	97	94	3
Mesothelioma [MESO]	87	87	0
Uveal Melanoma [UVM]	80	80	0
Adrenocortical carcinoma [ACC]	79	79	0
Kidney Chromophobe [KICH]	91	66	25
Uterine Corpus Endometrial Carcinoma [UCEC]	57	57	0
Diffuse Large B-cell Lymphoma [DLBC]	47	47	0
FFPE Pilot Phase II [FPPP]	45	45	0
Cholangiocarcinoma [CHOL]	45	36	9
Glioblastoma multiforme [GBM]	5	0	5
Total	9656	8993	663

## Results

**Fig. 1:**
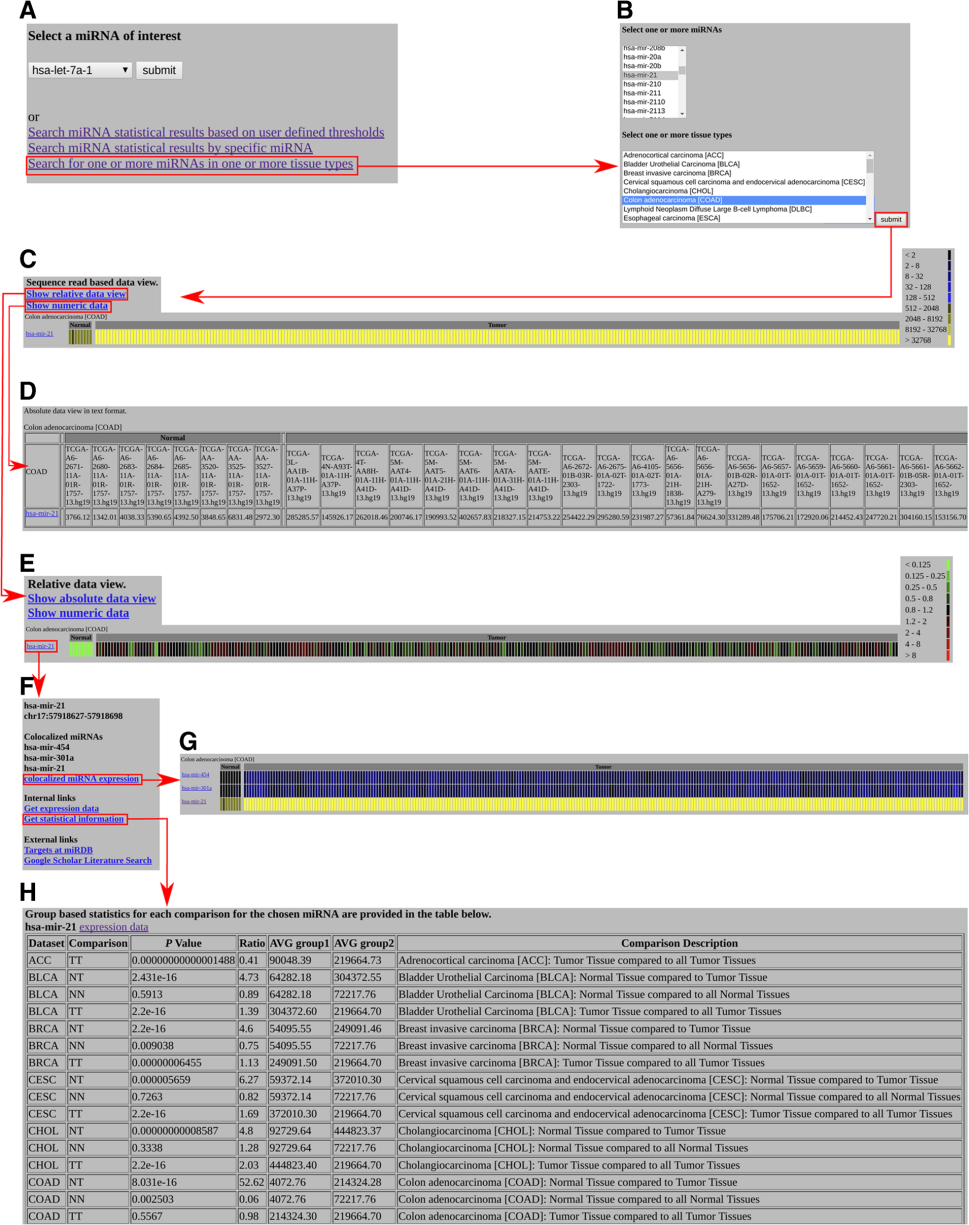
Screenshots of our sample analyses of miR-21 in COAD. **a**, **b** Advanced Search options in OMCD, enabling searches by miRNA, by cancer types, and by statistical results. **c** Heatmap. **d** Numeric view of absolute expressions of miR-21 in COAD control and tumor samples. **e** Heat map of relative expression of miR-21 in COAD. **f** Information and external links. **g** Heat map of miR-21 cluster members, enabling exploration of the expression patterns of colocalized miRNAs. **h** Statistical results of group-based comparisons of miR-21 in different cancer types

Our newly developed OMCD is available at www.oncomir.umn.edu/omcd. It features 4 types of search functions (Fig. 1a). For example, it currently includes miRNA expression data from 8 control colon tissue samples and 272 colon cancer (COAD) tumor samples. When we search for miR-21 in COAD samples (Fig. 1a, b), we obtain a heatmap showing the absolute expression level of miR-21 for all COAD samples (Fig. 1c). We can also obtain the numeric expression data (Fig. 1d; not completely shown, because of space limitations) and relative expression data (Fig. 1e). Clicking on hsa-miR-21 from the heatmap page, we are taken to a page showing links to additional analysis (Fig. 1f). These links provide detailed information about the chromosomal location of miR-21 and the names of colocalized miRNAs (miRNA clusters), as well as additional internal links to the expression data of miR-21 in other cancer types and to further statistical analysis (Fig. 1h).

In our COAD example, each miRNA specific OMCD webpage provides external links to the miRDB website for target prediction (www.mirdb.org) and to Google Scholar for literature searches [9]. From this webpage, we generate a link that allows the visualization of colocalized miRNA expression levels in a heatmap showing absolute expression (Fig. 1g). Expression levels of colocalized miRNAs can be displayed for all cancer types (not shown) and can be visualized in absolute and relative heatmaps as well as in the form of numeric data.

The 3 statistical analyses that we performed—using normal controls vs. tumor samples for each tumor type where available; tissue control samples vs. all other patients’ control samples; and each tumor sample type vs. all other tumor sample types—allowed us to visualize the expression patterns of miR-21 across different cancer types (Fig. 1h).

**Fig. 2:**
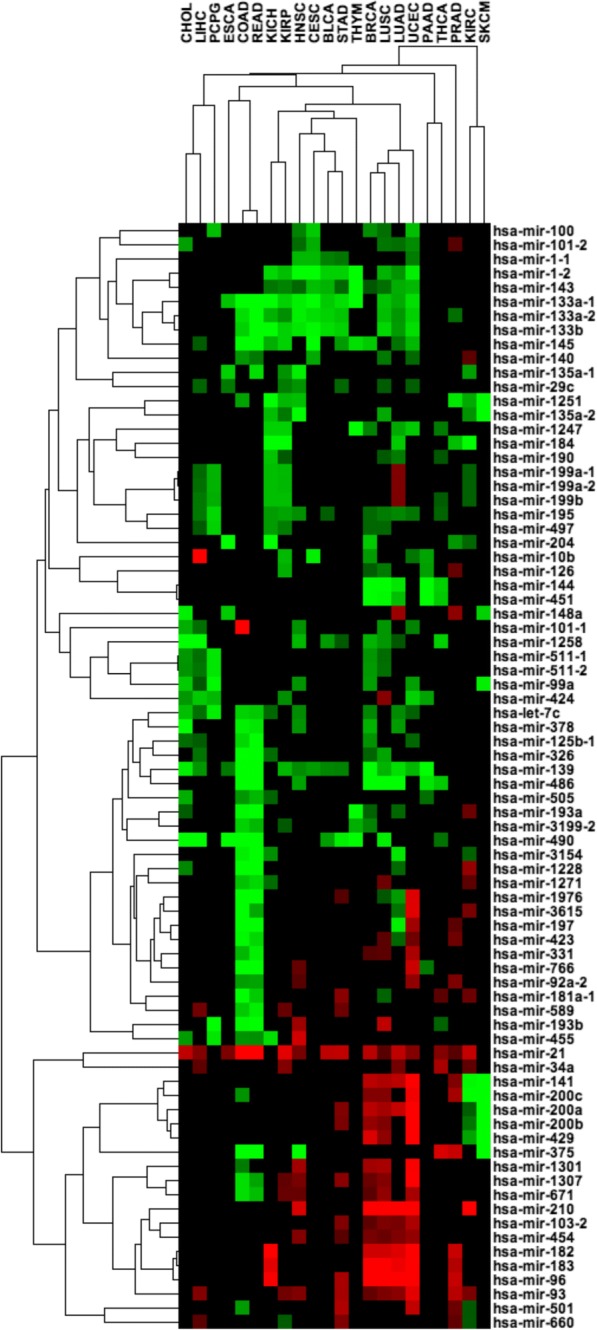
Heatmap of differentially expressed miRNAs in tumor vs. control samples (*P* < 0.000001, with a mean fold change in the tumor samples greater than 2 in 5 or more comparisons). Red = upregulation; green = downregulation

Further demonstrating OMCD’s utility, we were able to identify miRNAs that were recurrently differentially expressed between tumors and control samples. The difference was highly significant (*P* < 0.000001). In 5 such comparisons, the mean fold-change in the tumor samples was greater than 2 (Fig. 2). Many miRNAs are functionally well characterized and have been reported to be differentially expressed (between tumor and control samples) in a wide range of cancer types. For example, miR-21 is consistently upregulated in most cancer types [10]. Thus, it could potentially serve as cancer biomarker, but it may not be a suitable for identification of a *specific* cancer type. We were also able to observe decreases in miR-1/miR-133 in a number of cancers as well as gains in the miR-96/miR-182/miR-183 cluster in a number of other cancers.

In our OMCD testing, we also found that the COAD cluster and rectal cancer (READ) cluster had a very similar miRNA expression pattern, as compared with other cancer types. In COAD miR-101 showed higher expression levelsthen normal tissue and this increase was also observable in READ although not at the statistical power available for COAD.(Fig. 2).

Additionally, because the miR-101 expression was not significantly higher in other cancer types, it is reasonable to hypothesize that this miRNA is a biomarker for COAD. Similarly, we found that miR-10b expression was uniquely higher in hepatocellular carcinoma (LIHC), but not in other cancer types. These are a few examples of the testable hypotheses that OMCD can generate. To more thoroughly investigate the function of miR-21, mir-96/miR-182/miR-183 cluster in cancer, miR-101 in COAD, and miR-10b in LIHC, further experimental validation is warranted.

## Discussion

Evidence from the past decade indicates that miRNAs play a crucial role in the development of various cancer types. With the advent of high-throughput sequencing technology, more high-throughput miRNA expression data are now publicly available. Our OMCD database, developed at the University of Minnesota, is a simple web-based repository that allows easy and systematic comparative analyses of miRNA expression in various cancer types.

In our OMCD testing, we were able to identify increases in miR-101 as a biomarker candidate specifically for COAD. We found that its expression level was significantly higher in COAD tumors, but not in other tumors relative to normal samples. Previous studies, however, showed miR-101 expression levels in colorectal cancer that were different from our results [11, 12]. Those previous studies suggested that miR-101 expression was downregulated in colorectal cancer and that it was a tumor-suppressing miRNA whose overexpression inhibited tumor invasion and growth [11, 12].

Interestingly, when we used OncomiR (www.oncomir.org), which is also based on TCGA data, we again found that miR-101 was overexpressed in COAD tumors. Given the conflicting results for miR-101 in COAD in those 2 previous studies vs. our own use of both OMCD and OncomiR, further investigation into the function of miR-101 in COAD is needed, in order to definitively ascertain whether or not it is a suitable biomarker for COAD.

We also observed in our OMCD testing that miR-10b could be a potential biomarker for LIHC [13]. Previous studies showed that miR-10b was highly expressed in LIHC, that it was involved in neoplastic transformation of liver cancer stem cells, and that it promoted metastasis [14–16]. Other previous studies showed an oncogenic role of miR-10b in breast cancer, gastric cancer, and glioblastoma [17–20]. All of those studies suggest that miR-10b has a multifaceted function in many cancer types; further investigation is needed, in order to definitively ascertain whether or not it is a suitable biomarker for LIHC.

## Conclusions

Our current version of OMCD, derived from TCGA, contains the miRNA expression data of 9656 patients (represented by 8993 tumor samples and 663 control samples of normal tissue) with 33 different cancer types. To our knowledge, OncomiR (www.oncomir.org) is the only other TCGA-based online resource, besides OMCD, for analyzing miRNA expression data [5]. A limitation of both OncomiR and our current version of OMCD is their lack of miRNA datasets from other cancer patient cohorts. However, these were implemented in the miRGator 3.0 and miRCancerdb [6, 7]. But unlike OMCD, none of these databases have the option to analyze miRNA clusters. It is important to consider miRNA cluster members when studying miRNAs in cancers, especially to generate hypotheses from high-throughput data. Usually, miRNA cluster members have similar expression levels, but they potentially have vastly different biological functions. The ability to visualize and explore miRNA clusters in OMCD is crucial to develop defendable hypotheses.

In the future, we plan to expand OMCD by incorporating additional miRNA expression datasets from public data repositories such as Gene Expression Omnibus (GEO), Genomic Data Commons (GDC), and European Bioinformatics Institute (EBI). Doing so, we believe, will significantly improve the ability to use OMCD to develop defendable hypotheses.
